# The Short-Term and Long-Term Outcomes of Laparoscopy-Assisted Proximal Gastrectomy with Double-Tract Reconstruction versus Laparoscopy-Assisted Total Gastrectomy with Roux-en-Y Reconstruction for Adenocarcinoma of the Esophagogastric Junction: A Multicenter Study Based on Propensity Score Matching Analysis

**DOI:** 10.1155/2024/5517459

**Published:** 2024-05-27

**Authors:** Zhiwen Xu, Wei Lin, Su Yan, Shaoqin Chen, Jinping Chen, Qingqi Hong, Hexin Lin, Liangbin Xiao, Jingtao Zhu, Haoyu Bai, Xuejun Yu, Jun You

**Affiliations:** ^1^ Department of Gastrointestinal Oncology Surgery The First Affiliated Hospital of Xiamen University School of Medicine Xiamen University, Xiamen, China; ^2^ Department of Gastrointestinal Surgery The Affiliated Hospital of Putian College, Putian, China; ^3^ Department of Gastrointestinal Oncology Surgery The Affiliated Hospital of Qinghai University, Xining, China; ^4^ Department of Gastrointestinal Surgery The First Affiliated Hospital of Fujian Medical University, Fuzhou, China; ^5^ Department of Gastrointestinal Surgery The First Hospital of Quanzhou, Quanzhou, China

## Abstract

**Purpose:**

To compare the antireflux effect, long-term nutritional levels, and quality of life (QoL) between laparoscopy-assisted proximal gastrectomy with double-tract reconstruction (LPG-DTR) and laparoscopy-assisted total gastrectomy with Roux-en-Y reconstruction (LTG-RY) for adenocarcinoma of the esophagogastric junction (AEG).

**Methods:**

This multicenter retrospective cohort study collected clinicopathological and follow-up data of AEG patients from January 2016 to January 2021 at five high-volume surgery centers. The study included patients who underwent digestive tract reconstruction with LPG-DTR or LTG-RY after tumor resection. Propensity score matching (PSM) was utilized to minimize confounding factors. The comparison after PSM included postoperative complications, reflux esophagitis, long-term nutritional levels, and QoL.

**Results:**

A total of 151 consecutive patients underwent either LPG-DTR or LTG-RY. After PSM, 50 patients from each group were included in the analysis. The frequency of reflux esophagitis and Clavien–Dindo classification did not significantly differ between the two groups (*P* > 0.05). At 1 year after surgery, the LPG-DTR group showed significantly higher weight and hemoglobin levels than the LTG-RY group (*P* < 0.05). The overall postoperative Visick grade differed significantly between the groups (*P* < 0.05), but there was no significant difference in the proportion of patients with Visick≥III (*P* > 0.05).

**Conclusion:**

Both LPG-DTR and LTG-RY are safe and feasible methods for digestive tract reconstruction in patients with AEG. Both methods have similar antireflux effects and postoperative QoL. However, LPG-DTR resulted in superior nutritional levels compared to LTG-RY. Therefore, LPG-DTR is considered a relatively effective method for digestive tract reconstruction in AEG patients.

## 1. Introduction

The incidence of adenocarcinoma of the esophagogastric junction (AEG) has gradually increased in recent years, and its global incidence has been on the rise [1–4]. Due to its special location and different tumor biology [5, 6], the standardized treatment of AEG lacks a unified consensus, and there is no consensus on many issues. Proximal gastrectomy (PG) and total gastrectomy (TG) are both surgical approaches currently utilized in clinical practice. However, due to differences in the surgical techniques and extent of resection, these two procedures may exhibit significant differences in terms of antireflux effects, postoperative nutritional status, and quality of life (QoL) for patients [7, 8].

There are many methods of digestive tract reconstruction after PG, including esophagogastrostomy reconstruction, jejunal interposition reconstruction, double-tract reconstruction, tube-like stomach reconstruction, Kamikawa reconstruction, side overlap reconstruction, and various derived modified and compound techniques, each of which has its advantages and disadvantages [9–11]. However, it is unfortunate that there is still a lack of high-quality controlled studies comparing the aforementioned surgical approaches and total gastrectomy. There is still no consensus on the optimal surgical approach for AEG. Combining the clinicopathological and postoperative follow-up data of AEG patients admitted to five clinical centers in China in recent years, we compared and analyzed the laparoscopy-assisted proximal gastrectomy with double-tract reconstruction (LPG-DTR) and the laparoscopy-assisted total gastrectomy with Roux-en-Y reconstruction (LTG-RY) for the digestive tract, which is a promising approach for clinical use, using the propensity score matching (PSM) method. We aim to provide some evidence for clinical decision-making.

## 2. Materials and Methods

### 2.1. Study Design and Patient Population

From January 2016 to January 2021, patients with Siewert types II and III AEG who underwent surgical treatment at 5 institutions were enrolled in this multicenter cohort study. Based on the different extent of gastric resection and methods of digestive tract reconstruction, patients were divided into the LPG-DTR group and the LTG-RY group. PSM was performed to minimize potential confounding factors between the two groups. Some research methods in this study were referenced from our previous research design [12]. The study was approved by the Ethics Committee at the First Affiliated Hospital of Xiamen University (protocol code: XMYY-2022KY107).

### 2.2. Endpoints

The primary endpoints were the percentage of reflux esophagitis, long-term nutritional levels, and postoperative QoL in patients between the LPG-DTR and LTG-RY groups. The secondary endpoints were complications and surgical outcomes.

### 2.3. Inclusion Criteria

The inclusion criteria are as follows: (1) patients diagnosed with Siewert II and Siewert III AEG based on endoscopy and pathological biopsy, (2) patients who underwent LPG-DTR or LTG-RY, and (3) patients with no distant metastases detected on preoperative imaging.

### 2.4. Exclusion Criteria

The exclusion criteria are as follows: (1) patients who underwent laparotomy surgery, (2) patients with a history of malignancy, (3) patients who underwent combined organ resection, and (4) patients with preoperative symptoms of gastroesophageal reflux.

### 2.5. Surgical Procedure

All participating surgeons possess a high level of proficiency in laparoscopy-assisted gastrectomy procedures, having surpassed the learning curve associated with laparoscopy-assisted radical gastrectomy. The technical details of the digestive tract reconstruction procedures were previously described [12]. The patients were positioned in the supine position and administered general anesthesia. After pneumoperitoneum is established, the procedures of tissue and organ division, vascular ligation, and lymph node dissection are performed under laparoscopy. Subsequently, a 6-8 cm incision is made in the upper abdomen for extracorporeal gastrointestinal anastomosis. The LPG-DTR group underwent PG with D1+ lymph node dissection, while the LTG-RY group underwent LTG-RY with D2 lymph node dissection. The surgical procedures adhered to the instructions outlined in the Chinese consensus on surgical treatment for AEG and the guidelines provided by the Japanese Gastric Cancer Association [13, 14].

### 2.6. Evaluation Criteria

*Perioperative conditions*: operation time, blood loss, time to first flatus, days of abdominal drainage tube placement, dietary recovery time, postoperative hospitalization days, and postoperative complications were included in the patient characteristics.

*Postoperative complications*: postoperative complications include early complications and late complications. The grading of early complications is determined based on the Clavien–Dindo (CD) classification [15]. Early complications were defined as those occurring within 30 days after surgery, while those occurring thereafter were defined as late complications.

*Long-term conditions*: the occurrence of postoperative reflux esophagitis, anastomotic strictures, and other long-term postoperative complications in patients; changes in nutritional indicators such as weight, hemoglobin, and albumin. Reflux esophagitis was assessed by a combination of endoscopy and the GerdQ scale at 1 year after operation [16, 17].

*Postoperative QoL evaluation*: the Visick grade was used to assess patients' QoL 1 year after surgery. It was divided into four grades: Visick I: excellent recovery after surgery, no symptoms; Visick II: occasional discomforts such as epigastric fullness, diarrhea, or other mild symptoms, which do not affect daily life and work; Visick III: mild to moderate symptoms, such as dumping syndrome, reflux esophagitis, and other symptoms requiring medication and intervention, and patients can live and work normally; Visick IV: moderate to severe symptoms or obvious complications, patients are unable to live and work normally. The higher the grade, the greater the degree of discomfort in daily life after surgery and the poorer the QoL [18].

### 2.7. Follow-Up

All patients received follow-up by telephone, mail, or outpatient visits. The re-examinations mainly included laboratory blood tests, computed tomography scans, and physical examinations. Endoscopy was recommended once a year after the operation. Patients were followed up regularly every 6 months for the first 2 years and then annually until 5 years postoperatively.

### 2.8. Statistical Analysis

We conducted all statistical analyses using the SPSS 26.0 statistical software. PSM was based on sex, age, height, weight, BMI, preoperative glucose, preoperative hemoglobin, preoperative total protein, preoperative albumin, preoperative neoadjuvant radiotherapy, tumor length diameter, and TNM stage and involved 1 : 1 nearest neighbor matching using a caliper of 0.15. Continuous variables were evaluated using Student's *t*-test. Skewed data were reported as *M* (range), and between-group differences were analyzed using nonparametric tests. Categorical data were presented as frequencies and percentages, and intergroup comparisons were conducted using appropriate tests such as the chi-square test, Fisher's exact probability method, or Mann–Whitney *U* test, depending on the distribution of the data. All statistical tests were two tailed, and statistical significance was set at *P* < 0.05.

## 3. Results

### 3.1. Baseline Information before and after PSM

**Figure 1:**
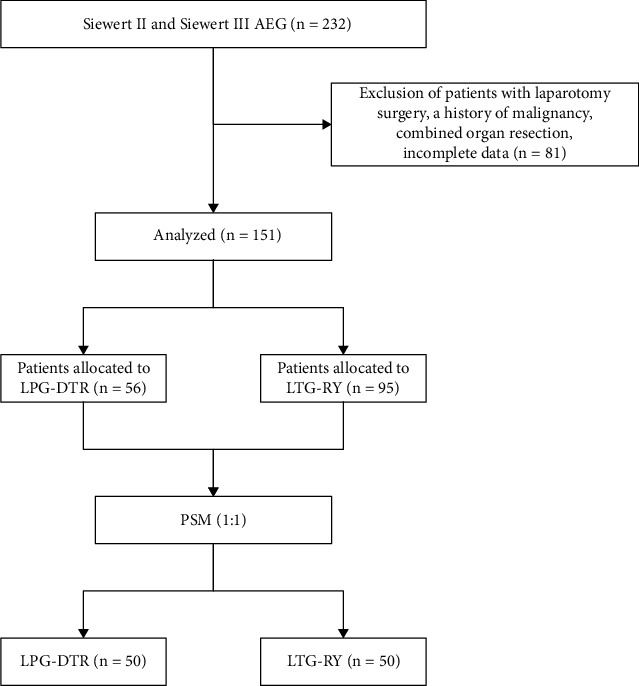
Consort diagram of this study. AEG = adenocarcinoma of the esophagogastric junction; LPG-DTR = laparoscopy-assisted proximal gastrectomy with double-tract reconstruction; LTG-RY = laparoscopy-assisted total gastrectomy with Roux-en-Y reconstruction; PSM = propensity score matching.

The consort diagram of this study is shown in Figure 1. A total of 151 consecutive patients who underwent LPG-DTR or LTG-RY were included according to the predetermined inclusion and exclusion criteria. The sample comprised 90 patients from the First Affiliated Hospital of Xiamen University, 38 patients from the Affiliated Hospital of Putian College, 14 patients from the Affiliated Hospital of Qinghai University, 4 patients from the First Affiliated Hospital of Fujian Medical University, and 5 patients from the First Hospital of Quanzhou, all of whom underwent successful surgery with R0 resection and no perioperative deaths.

Tables 1 and 2 present the baseline characteristics of patients both before and after PSM. Out of the 151 patients included in the study, 56 underwent LPG-DTR and 95 underwent LTG-RY. Following PSM, 50 patients were successfully matched and included in the analysis for each group. After PSM, no significant differences were observed in the baseline parameters between the two groups (*P* > 0.05), suggesting that the groups were comparable.

### 3.2. Intraoperative and Postoperative Conditions

Table 3 provides the perioperative outcomes. Following PSM, there were no significant differences observed between the LPG-DTR and LTG-RY groups regarding the operation time, intraoperative blood loss, duration of abdominal drainage tube placement, time to first flatus, postoperative dietary recovery time, and postoperative hospitalization days (*P* > 0.05). However, the LPG-DTR group exhibited significantly fewer total harvested lymph nodes and positive lymph nodes compared to the LTG-RY group (*P* < 0.05).

### 3.3. Postoperative Complications

The postoperative complications are outlined in Table 4. The overall incidence of early complications was 32.0% (16/50) in both groups after PSM. In the LPG-DTR group, the early complications were CD grades I, II, III, IV, and V in 2 (4%), 10 (20%), 4 (8%), 0 (0%), and 0 (0%) patients, respectively. Among these patients, there were 6 cases of pulmonary infection, 6 cases of pleural effusion, 2 cases of incisional infection, and 3 cases of anastomotic fistula. In the LTG-RY group, the early complications were CD grades I, II, III, IV, and V in 0 (0%), 11 (22%), 5 (10%), 0 (0%), and 0 (0%) patients, respectively. These patients included 10 cases of pulmonary infection, 9 cases of pleural effusion, 0 cases of incisional infection, and 3 cases of anastomotic fistula. There were no CD grade IV or V patients in either group. Furthermore, there was no significant difference in the CD classification of early complications between the two groups (*P* > 0.05). Early complications were dominated by pulmonary complications in both groups. All early complications experienced by any patient were successfully treated prior to discharge.

Regarding late complications, 6 cases of reflux esophagitis developed in the LPG-DTR group and 5 cases in the LTG-RY group, but the difference between the two groups did not reach statistical significance (12% versus 10%, respectively; *P* = 1.000). There were 3 cases of anastomotic stricture in the LPG-DTR group and 1 case in the LTG-RY group, with incidence rates of 6% and 2%, respectively, but the differences between the two groups were not statistically significant. Postoperative intestinal obstruction occurred in 2 cases in both the LPG-DTR group and the LTG-RY group, resulting in an incidence rate of 4% in both groups.

### 3.4. Nutrition-Related Parameters

**Figure 2:**
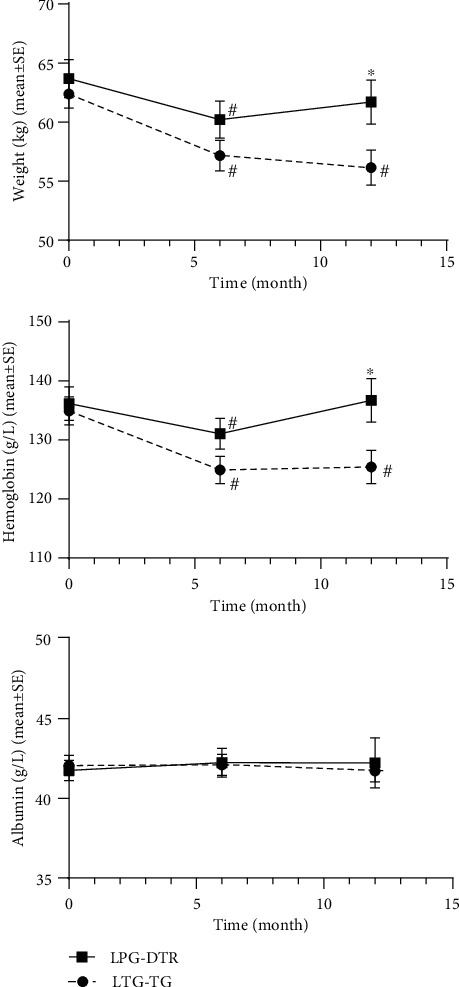
Nutritional indicators of LPG-DTR and LTG-RY after PSM (weight, hemoglobin, and albumin). LPG-DTR = laparoscopy-assisted proximal gastrectomy with double-tract reconstruction; LTG-RY = laparoscopy-assisted total gastrectomy with Roux-en-Y reconstruction. ^∗^*P* less than 0.05 between two groups. ^#^*P* less than 0.05 comparison with preoperative.

Table 5 and Figure 2 present the comparison of nutritional parameters among different surgical strategies. The changes in nutritional indicators between the two groups at 6 months and 1 year postoperatively were analyzed. At 1 year postoperatively, weight levels were significantly higher in the LPG-DTR group compared to the LTG-RY group (*P* < 0.05), and hemoglobin levels were also significantly superior in the LPG-DTR group (*P* < 0.05). However, there was no statistical difference in albumin levels between the two groups at 6 months and 1 year postoperatively (*P* > 0.05).

In the LPG-DTR group, weight significantly decreased at 6 months postoperatively compared to preoperative levels. However, there was no significant difference at 1 year postoperatively, indicating weight restoration to preoperative levels. Hemoglobin levels were significantly lower at 6 months postoperatively, but not significantly different at 1 year postoperatively. Albumin levels showed no significant difference at 6 months and 1 year postoperatively.

In the LTG-RY group, weight significantly declined at both 6 months and 1 year postoperatively compared to preoperative levels. Hemoglobin levels showed significant decreases at both 6 months and 1 year postoperatively. Albumin levels did not change significantly at 6 months and 1 year postoperatively.

### 3.5. Postoperative Quality of Life

Table 6 presents the results of the Visick grade. In the LPG-DTR group, 48% (24/50) were classified as Visick I, 36% (18/50) as Visick II, 16% (8/50) as Visick III, and 0% (0/50) as Visick IV. In the LTG-RY group, 74% (37/50) were Visick I, 12% (6/50) were Visick II, 14% (7/50) were Visick III, and 0% (0/50) was Visick IV. There was a statistically significant difference in the overall Visick grade between the two groups (*P* < 0.05). When comparing patients classified as Visick≤II versus Visick≥III, there was no significant difference in the proportion of patients with Visick≥III between the two groups (*P* > 0.05). Both groups consisted mainly of Visick I and II patients, indicating mild or no symptoms without the need for additional medical intervention.

## 4. Discussion

In PG, esophagogastrostomy has been widely used in clinical practice due to its simplicity of operation and adherence to a near-normal digestive tract structure. However, many surgeons have gradually discovered that although this procedure preserves a portion of the stomach, many patients experience severe gastroesophageal reflux symptoms after surgery, significantly reducing their postoperative QoL, thus outweighing the benefits. For patients with AEG, the ideal situation would be to preserve a portion of the stomach while minimizing the occurrence of postoperative gastroesophageal reflux. Therefore, in the context of PG, the design of an antireflux mechanism becomes particularly important.

In an endeavor to strike a balance between postoperative nutrition and QoL, numerous surgeons have explored various antireflux strategies [19–22]. Among the different methods of digestive tract reconstruction, double-tract reconstruction following proximal gastrectomy has gained traction in select medical centers in recent years. Retrospective studies have indicated that this approach exhibits superior antireflux effects compared to esophagogastrostomy reconstruction, positioning it as a promising reconstruction technique [23–25]. In this study, we undertook a comparative analysis of different surgical approaches in patients with AEG, with the primary aim of evaluating the antireflux effects, postoperative nutritional status, and QoL outcomes associated with LPG-DTR and LTG-RY procedures.

In terms of perioperative complications, there was no significant difference between the two groups in most of the early postoperative indicators, such as pulmonary infection, pleural effusion, and incisional infection. This is similar to the results of the KLASS05 study in Korea [26]. Since only D1+ lymph node dissection was required in the LPG-DTR group, while the standard D2 lymph node dissection was performed in the LTG-RY group, the total number of lymph nodes dissected was significantly higher than that in the PG group, and thus, more positive lymph nodes were detected. Three cases of anastomotic fistula occurred in both groups after matching, further indicating that LPG-DTR and LTG-RY were safe and feasible.

After PSM, the occurrence rates of reflux esophagitis were found to be 12% and 10% in the LPG-DTR and LTG-RY groups, respectively, with no significant difference observed between the two groups. Recent studies have shown that the incidence of reflux esophagitis within the first year after double-tract reconstruction is merely 12%, which is significantly lower compared to esophagogastrostomy reconstruction [27, 28]. In a separate controlled study conducted by Japanese researchers comparing double-tract reconstruction with esophagogastrostomy reconstruction, the incidence of reflux esophagitis after double-tract reconstruction was determined to be 12.5% [29]. Gastroesophageal reflux represents a critical factor influencing the postoperative QoL in PG patients. The loss of natural antireflux structures such as the lower esophageal sphincter and gastric fundus resulting from PG is the primary cause of severe reflux in individuals undergoing esophagogastrostomy.

In order to minimize the significant impact of postoperative reflux symptoms on patients' normal life, many medical centers opt for TG in patients with AEG. As the stomach is an important digestive organ in the human body, TG inevitably affects the digestion and absorption of food in patients after surgery. Double-tract reconstruction, by placing a 10-15 cm segment of jejunum between the remnant stomach and the esophagus, extends the distance of refluxed digestive juices into the esophagus, thereby reducing the incidence of reflux esophagitis [30].

Based on current clinical research reports, Kamikawa is considered one of the most effective digestive tract reconstruction procedures for antireflux effects in the context of PG. In a retrospective study including 546 patients, the incidence of reflux esophagitis after Kamikawa reconstruction was 10.6% [31], whereas the reflux rate of LPG-DTR in our study was 12.0%, which further indicates that LPG-DTR is not significantly inferior to Kamikawa reconstruction in terms of antireflux effect [32].

The LPG-DTR preserves two food digestion pathways. One part of the food enters the jejunum directly from the esophagus, and the other part enters the jejunum via the residual stomach and duodenal pathway for digestion and absorption, which not only facilitates the absorption of nutrients but also promotes the secretion of gastrointestinal hormones [33]. In our study, patients in the LPG-DTR group demonstrated significantly higher levels of body weight and hemoglobin 1 year after surgery compared to the LTG-RY group. The LPG-DTR group not only exhibited favorable effects on weight maintenance but also reduced the occurrence of postoperative anemia, which aligns with the findings of a meta-analysis involving 4407 patients [34]. LPG-DTR offers advantages in postoperative nutrition compared to LTG-RY.

At present, there are still some doubts about the extent to which the residual gastric channel can act for food diversion after double-tract reconstruction [35]. And some operators have also observed that the contrast agent does not pass through the residual gastric channel during the follow-up period. The long-term practical effects of the two pathways in the double tract are still worth continued attention.

Based on our research findings, two crucial purposes of LPG-DTR are evident. Firstly, LPG-DTR preserves a portion of the stomach, leading to improved nutritional levels. Secondly, our research indicates that LPG-DTR can provide antireflux effects that are comparable to those of LTG-RY and are not inferior to them.

In this study, we also conducted a comparative analysis of postoperative QoL between the two groups of patients. The QoL of patients is influenced by various factors, including gastroesophageal reflux, anastomotic stenosis, and dumping syndrome. Among these, gastroesophageal reflux is an important factor affecting patients' postoperative QoL [36, 37]. The LPG-DTR provides a better antireflux effect, and the patient's postoperative QoL will have a greater improvement compared to patients with esophagogastrostomy. The majority of patients with LPG-DTR have an acceptable postoperative QoL. Our results on QoL are close to those of some recent studies [38].

As a procedure that has evolved from Roux-en-Y reconstruction, double-tract reconstruction is relatively easy to cross the learning curve and is conducive to widespread implementation in clinical practice. If the Kamikawa technique is not mature, double-tract reconstruction serves as a viable alternative to TG. However, it should be noted that double-tract reconstruction involves multiple anastomoses, and manual reinforcement is necessary after stapler anastomosis.

## 5. Conclusion

Both LPG-DTR and LTG-RY are safe and feasible methods for digestive tract reconstruction in patients with AEG. Both methods have similar antireflux effects and postoperative QoL. The majority of patients in both groups reported satisfactory and acceptable QoL. However, patients who underwent LPG-DTR had better nutritional levels than those who underwent LTG-RY. LPG-DTR is a relatively effective method for digestive tract reconstruction in patients with AEG. Due to the limited sample size and follow-up duration in our study, further validation of these findings and oncological outcomes requires larger sample sizes and higher-quality prospective studies.

## Figures and Tables

**Table 1:** Basic characteristics of the patients before PSM.

	LPG-DTR (*n* = 56)	LTG-RY (*n* = 95)	*P* value
Age (years)	62.0 ± 9.0	60.8 ± 8.0	0.413
Sex			0.890
Female	13	23	
Male	43	72	
Height (cm)	166.9 ± 7.8	163.7 ± 9.8	0.038
Weight (kg)	63.8 ± 11.1	61.0 ± 9.2	0.302
Preoperative BMI (kg/m^2^)	22.9 ± 3.9	22.7 ± 3.8	0.958
Glucose (*μ*mol/L)	5.3 ± 1.1	5.5 ± 1.4	0.023
Hemoglobin (g/L)	136.4 ± 19.2	129.5 ± 19.1	<0.001
Total protein (g/L)	69.8 ± 7.9	70.1 ± 7.6	0.314
Albumin (g/L)	41.7 ± 4.5	41.3 ± 4.7	0.025
Tumor size (cm)	2.4 ± 1.0	3.7 ± 1.9	<0.001
pTNM stage			
I	41	40	<0.001
II	9	23	
III	6	32	
Neoadjuvant chemoradiotherapy	1	18	0.055

Data are shown as mean ± SD or number. LPG-DTR = laparoscopy-assisted proximal gastrectomy with double-tract reconstruction; LTG-RY = laparoscopy-assisted total gastrectomy with Roux-en-Y reconstruction. TNM staging was performed according to the AJCC 7th edition.

**Table 2:** Basic characteristics of the patients after PSM.

	LPG-DTR (*n* = 50)	LTG-RY (*n* = 50)	*P* value
Age (years)	61.5 ± 8.9	62.4 ± 6.3	0.587
Sex			0.629
Female	12	10	
Male	38	40	
Height (cm)	166.5 ± 7.8	166.9 ± 8.06	1.000
Weight (kg)	63.7 ± 11.4	62.6 ± 11.40	0.514
Preoperative BMI (kg/m^2^)	23.0 ± 4.0	22.5 ± 3.12	0.466
Glucose (*μ*mol/L)	5.3 ± 1.2	5.4 ± 1.20	0.662
Hemoglobin (g/L)	136.2 ± 20.1	134.5 ± 18.14	0.739
Total protein (g/L)	70.0 ± 8.3	69.49 ± 7.3	0.764
Albumin (g/L)	41.8 ± 4.5	41.2 ± 4.17	0.739
Tumor size (cm)	2.5 ± 1.0	2.83 ± 1.40	0.196
pTNM stage			
I	35	28	0.086
II	9	8	
III	6	14	
Neoadjuvant chemoradiotherapy	1	2	1.000

Data are shown as mean ± SD or number. LPG-DTR = laparoscopy-assisted proximal gastrectomy with double-tract reconstruction; LTG-RY = laparoscopy-assisted total gastrectomy with Roux-en-Y reconstruction. TNM staging was performed according to the AJCC 7th edition.

**Table 3:** Surgical outcomes after PSM.

	LPG-DTR (*n* = 50)	LTG-RY (*n* = 50)	*P* value
Operation time (min)	264.4 ± 63.1	270.1 ± 63.3	0.661
Intraoperative blood loss (mL)	94.9 ± 113.2	115.3 ± 92.6	0.326
Time to first flatus after surgery (days)	3.5 ± 2.0	4.6 ± 3.7	0.077
Removal of abdominal drainage (days)	8.6 ± 6.4	9.8 ± 6.5	0.380
Time to first soft diet (days)	7.4 ± 3.9	7.4 ± 3.9	0.463
Postoperative hospital stay (days)	15.3 ± 9.1	13.9 ± 8.9	0.438
Number of retrieved lymph nodes	20.8 ± 9.7	38.2 ± 15.1	<0.001
Number of positive lymph nodes	0.5 ± 1.5	3.0 ± 5.0	0.002

Data are shown as mean ± SD or number. LPG-DTR = laparoscopy-assisted proximal gastrectomy with double-tract reconstruction; LTG-RY = laparoscopy-assisted total gastrectomy with Roux-en-Y reconstruction.

**Table 4:** Postoperative complications after PSM.

	LPG-DTR (*n* = 50)	LTG-RY (*n* = 50)	*P* value
Early complications			
No	34 (68.0)	34 (68.0)	
Yes	16 (32.0)	16 (32.0)	
Pulmonary infection	6 (12.0)	10 (20.0)	0.275
Pleural effusion	6 (12.0)	9 (18.0)	0.401
Abdominal incision infection	2 (4.0)	0 (0.0)	0.495
Anastomotic leakage	3 (6.0)	3 (6.0)	1.000
Clavien–Dindo classification			
Grade I	2 (4.0)	0 (0.0)	0.391
Grade II	10 (20.0)	11 (22.0)	
Grade III	4 (8.0)	5 (10.0)	
Grade IV	0 (0)	0 (0)	
Grade V	0 (0)	0 (0)	
Late complications			
No	41 (82.0)	42 (84.0)	
Yes	9 (18.0)	8 (16.0)	
Reflux esophagitis	6 (12.0)	5 (10.0)	1.000
Anastomotic stenosis	3 (6.0)	1 (2.0)	0.617
Intestinal obstruction	2 (4.0)	2 (4.0)	1.000

Data are shown as number (%). LPG-DTR = laparoscopy-assisted proximal gastrectomy with double-tract reconstruction; LTG-RY = laparoscopy-assisted total gastrectomy with Roux-en-Y reconstruction.

**Table 5:** Nutritional indicators after PSM.

	LPG-DTR (*n* = 50)	LTG-RY (*n* = 50)	*P* value
Weight (kg)			
Preoperative	63.7 ± 11.4	62.4 ± 8.4	0.514
6 months after operation	60.2 ± 10.2	57.2 ± 8.5	0.135
1 year after operation	61.7 ± 12.6	56.1 ± 8.4	0.031
Hemoglobin (g/L)			
Preoperative	136.2 ± 20.1	134.9 ± 16.8	0.739
6 months after operation	131.1 ± 15.4	124.9 ± 15.1	0.081
1 year after operation	136.7 ± 18.2	125.4 ± 17.0	0.017
Albumin (g/L)			
Preoperative	41.8 ± 4.5	42.1 ± 4.5	0.739
6 months after operation	42.2 ± 5.4	42.1 ± 4.3	0.901
1 year after operation	42.2 ± 7.7	41.7 ± 4.0	0.741

Data are shown as mean ± SD or number. LPG-DTR = laparoscopy-assisted proximal gastrectomy with double-tract reconstruction; LTG-RY = laparoscopy-assisted total gastrectomy with Roux-en-Y reconstruction.

**Table 6:** The Visick grade of the two groups after PSM.

	LPG-DTR (*n* = 50)	LTG-RY (*n* = 50)	*P* value
I	24 (48%)	37 (74%)	0.023
II	18 (36%)	6 (12%)	
III	8 (16%)	7 (14%)	
IV	0 (0%)	0 (0%)	
≥III	8 (16%)	7 (14%)	0.779

Data are shown as number (%). LPG-DTR = laparoscopy-assisted proximal gastrectomy with double-tract reconstruction; LTG-RY = laparoscopy-assisted total gastrectomy with Roux-en-Y reconstruction.

## Data Availability

The datasets used and/or analyzed during the current study are available from the corresponding author on reasonable request.
